# Integrated Community Profiling Indicates Long-Term Temporal Stability of the Predominant Faecal Microbiota in Captive Cheetahs

**DOI:** 10.1371/journal.pone.0123933

**Published:** 2015-04-23

**Authors:** Anne A. M. J. Becker, Geert P. J. Janssens, Cindy Snauwaert, Myriam Hesta, Geert Huys

**Affiliations:** 1 Laboratory of Microbiology, Department of Biochemistry and Microbiology, Faculty of Sciences, Ghent University, Ghent, Belgium; 2 BCCM/LMG Bacteria Collection, Department of Biochemistry and Microbiology, Faculty of Sciences, Ghent University, Ghent, Belgium; 3 Laboratory of Animal Nutrition, Department of Nutrition, Genetics and Ethology, Faculty of Veterinary Medicine, Ghent University, Merelbeke, Belgium; University of Illinois, UNITED STATES

## Abstract

Understanding the symbiotic relationship between gut microbes and their animal host requires characterization of the core microbiota across populations and in time. Especially in captive populations of endangered wildlife species such as the cheetah (*Acinonyx jubatus*), this knowledge is a key element to enhance feeding strategies and reduce gastrointestinal disorders. In order to investigate the temporal stability of the intestinal microbiota in cheetahs under human care, we conducted a longitudinal study over a 3-year period with bimonthly faecal sampling of 5 cheetahs housed in two European zoos. For this purpose, an integrated 16S rRNA DGGE-clone library approach was used in combination with a series of real-time PCR assays. Our findings disclosed a stable faecal microbiota, beyond intestinal community variations that were detected between zoo sample sets or between animals. The core of this microbiota was dominated by members of *Clostridium* clusters I, XI and XIVa, with mean concentrations ranging from 7.5-9.2 log10 CFU/g faeces and with significant positive correlations between these clusters (P<0.05), and by *Lactobacillaceae*. Moving window analysis of DGGE profiles revealed 23.3-25.6% change between consecutive samples for four of the cheetahs. The fifth animal in the study suffered from intermediate episodes of vomiting and diarrhea during the monitoring period and exhibited remarkably more change (39.4%). This observation may reflect the temporary impact of perturbations such as the animal’s compromised health, antibiotic administration or a combination thereof, which temporarily altered the relative proportions of *Clostridium* clusters I and XIVa. In conclusion, this first long-term monitoring study of the faecal microbiota in feline strict carnivores not only reveals a remarkable compositional stability of this ecosystem, but also shows a qualitative and quantitative similarity in a defined set of faecal bacterial lineages across the five animals under study that may typify the core phylogenetic microbiome of cheetahs.

## Background

As first shown in human studies and later also confirmed for other animal species, diet is considered a major regulator of the intestinal microbial composition and metabolic homeostasis in all mammals [1]. Also in zoo animal husbandry, nutrition is one of the most critical components [2], to the extent that feeding mismanagement is recognized as a major risk factor for suboptimal health, low breeding performance and a higher incidence of gastrointestinal and metabolic diseases [3,4]. The latter is especially true for endangered strict carnivores such as the cheetah (*Acinonyx jubatus*) where high vulnerability to gastrointestinal disorders in captivity is a major concern [5,6].

Conservation programs would clearly benefit from a finer scale understanding of the relationship between food availability (e.g. fasting frequency), gut physiology and symbiotic gut microbes in cheetahs [7–9]. Also, such information may prove to be a key component to enhance feeding strategies for disease prevention or therapeutic intervention [10–12]. Due to its close phylogenetic relationship and the fact that it is a far better studied and accessible species, the domestic cat has been proposed as reference model for dietary intervention studies in exotic wild felids kept under human care such as cheetahs [13]. However, the scientific rationale of this proposal is questionable because dietary regimes and feeding habits of the domestic cat and wild feline species are markedly different, and this is likely to affect the taxonomic structure of their gastrointestinal (GI) microbiota as well. In fact, a recent study characterizing the faecal microbiota of two cheetahs under care in a Belgian zoo revealed a pronounced underrepresentation of *Bifidobacteriaceae* and Bacteroidetes members compared to domestic cats [14]. These data thus highlight the need for a more advanced and fundamental understanding of the cheetah’s GI microbiota itself instead of focussing on extrapolation from genetically related species that evolved away from a strict carnivorous diet as a result of domestication. However, before specific dietary applications can be developed in any population of captive cheetahs on the long term, it is of particular relevance to assess the cross-sectional and longitudinal variation within their intestinal ecosystem and to identify its potential core members [15,16].

Recently, we provided a first taxonomic baseline of the predominant faecal bacterial populations based on single samples of two captive cheetahs from the same zoo [14]. The aim of this follow-up study was to address the temporal stability of the intestinal microbiota of captive cheetahs by including more animals from different zoos sampled at multiple time points. To this end, we carried out a longitudinal study with bi-monthly faecal sampling of 5 cheetahs housed in two European zoos over a 3-year period. An integrated 16S rRNA DGGE-clone library approach was used in combination with real-time PCR assays to monitor microbial community variation and diversity in the time series samples.

## Methods

### Ethics statement

This study was conducted non-invasively, without animal handling or any change in daily management and housing conditions of the animals. Faecal samples were collected from two adult cheetahs housed at Zoo Parc Planckendael (Belgium) and three adult cheetahs housed at Zoo Parc Overloon (The Netherlands) under supervision of the zookeepers. Both zoos are recognized members of the European Association of Zoos and Aquaria (EAZA) and the cheetahs are housed according to the Minimum Standards for the Accommodation and Care of Animals in Zoos and Aquaria and the EAZA Code of Practice. Permission for faecal sampling was obtained from the staff veterinarians and mammal curators. Ghent University Animal Ethics Committee approval and any additional permits were not further needed.

### Animals & Diet

Five adult cheetahs were included from two institutions that are full members of the European Association of Zoos and Aquaria (EAZA, http://www.eaza.net/membership). At Zoo Parc Planckendael (PL; 50° 99’ N, 4° 52’ E) in Belgium, the two male cheetahs (B1 and B2; born in 2001) shared indoor and outdoor housing and were fed their regular zoo diet consisting of chunked boneless horsemeat (2 kg/day/animal) topdressed with a vitamin and mineral premix (Carnicon; Aveve, Leuven, Belgium) and sometimes interspersed with unsupplemented whole rabbits. No medical or health problems were reported or apparent on remote examination, and both cheetahs were treated prophylactically for internal parasites (Horseminth; Pfizer, Brussels, Belgium) approximately every two months during the entire study period starting one week before first sampling (PL-T1).

At Zoo Parc Overloon (OV; 51° 57’ N, 5° 94’ E) in the Netherlands, the two male cheetahs (NL9 and NL10; respectively born in 2000 and 2002) shared indoor and outdoor housing whereas the female cheetah (NL11; born in 2005) was housed separately. During the study, animal NL11 gave birth to four cubs. From sampling point OV-T4 onwards, NL11 shared indoor and outdoor housing with her litter of which two survived the following year. All cheetahs from OV were fed their regular zoo diet consisting of unsupplemented whole rabbits randomly interspersed with chicken, vitamin and mineral supplemented chunked boneless horsemeat (1.5 kg/day/animal) or pieces of beef, horse and antilope carcasses. The three cheetahs were treated prophylactically roughly on a three monthly basis for internal parasites (Drontal Cat; Bayer, Mijdrecht, the Netherlands), *i*.*e*. one month before first sampling OV-T1, between OV-T2 & OV-T3, OV-T5 & OV-T6, OV-T10 & OV-T11, OV-T11 & OV-T12 and OV-T12 & OV-T13. Interim vomiting, diarrhea, loss of appetite and overall weakness were reported for animal NL9 between the time of sampling points OV-T2 and OV-T5. Both male cheetahs were also relocated and left the study at sampling point OV-T11.

### Sample collection

Over a 3-year period starting in October 2010 in PL and May 2011 in OV, cheetahs were bimonthly monitored for 12h to collect animal-specific faecal samples immediately upon defaecation. This way, a total of 55 fresh faecal samples (100–200 gram/animal) were collected. At PL, monitoring during 16 different time points (PL-T1 to PL-T16) resulted in 14 samples each from animals B1 and B2. At OV, 8 faecal samples were collected from animal NL9, 9 from animal NL10 and 10 from animal NL11 at 14 different time points (OV-T1 to OV-T14). For a number of time points samples are missing because no defaecation was observed. All samples were scored for consistency [17], aliquoted into plastic tubes, transported on dry ice and stored at -80°C until further analysis. In addition, each animal’s medical history and the dietary regime *i*.*e*. the diet type (type of meat or prey) and fasting days were recorded on a daily basis. At PL, 14 samples were collected from animals B1 and B2 at 16 different time points (PL-T1 to PL-T16). At OV, 8 faecal samples were collected from animal NL9, 9 from animal NL10 and 10 from animal NL11 at 14 different time points (OV-T1 to OV-T14).

### DNA extraction

Prior to DNA extraction, 25 grams (wet weight) of each thawed faecal sample was placed separately in sterile stomacher bags and homogenized in 225 ml peptone-buffered saline (PBS) (0.1% [wt/vol] bacteriological peptone [L37; Oxoid, Basingstoke, United Kingdom], 0.85% [wt/vol] NaCl [106404; Merck, Darmstadt, Germany]). The sludgy homogenate was filtered on a Büchner funnel to discard large particles such as hair and bones, and subsequently divided into 1.5 ml aliquots which were stored at -80°C.

The protocol of Pitcher et al. [18] was used in a modified version [19] to extract total bacterial DNA from the faecal samples. DNA size and integrity were assessed on 1% agarose electrophoresis gels stained with ethidium bromide. DNA concentration and purity were determined by spectrophotometric measurements at 234, 260 and 280 nm. DNA extracts were finally diluted to OD 1 with TE buffer (1 mM EDTA [324503; Merck, Darmstadt, Germany], 10 mM Tris-HCl [648317; Merck, Darmstadt, Germany]) and stored at -20°C.

### Community PCR for Denaturing Gradient Gel Electrophoresis (DGGE)

The variable V3 region of the 16S rRNA gene was amplified using the universal bacterial primers GC-F357 (5’-CCTACGGGAGGCAGCAG-3’) and R518 (5’-ATTACCGCGGCTGCTGG-3’) [20,21]. PCR was performed with a *Taq* polymerase kit (Applied Biosystems, Gent, Belgium). The PCR reaction mix included: 6 μl 10x PCR buffer (containing 15 mM MgCl_2_), 2.5 μl bovine serum albumin (0.1 mg/ml), 2.5 μl 2 mM dNTPs, 2 μl of each primer (5 μM), 0.25 μl *Taq* polymerase, 33.75 μl milliQ water and 1 μl 10-fold diluted DNA solution. All reactions were carried out in a final volume of 50 μl using the following PCR programme: initial denaturation at 95°C for 1 min, 30 cycles of 95°C for 30 s, 55°C for 45 s and 72°C for 1 min, and final extension of 72°C for 7 min. Negative (milliQ water as template) and positive controls were included in parallel. Amplicons were checked on a 1% agarose gel under UV illumination after ethidium bromide staining. PCR amplification products were stored at -20°C until DGGE analysis.

### DGGE analysis and gel processing

The resulting 16S rRNA amplicons were analyzed with DGGE fingerprinting (D-code System, Bio-Rad, Nazareth, Belgium) using a 35–70% denaturing gradient as previously described [21]. Per lane, 25 μl of PCR product was loaded and electrophoresis was performed in 1x Tris-Acetate-EDTA buffer (TAE, catalog no. 161–077, Bio-Rad) for 990 min at a constant voltage of 770 V. Afterwards, DGGE gels were stained for 30 min with 1x SYBR Gold nucleic acid gel stain (S-11494, Invitrogen, Merelbeke, Belgium) and band profiles were visualized using a charge-coupled device (CCD) camera and the Bio-Rad Quantity One software program.

A standard reference lane containing the V3-16S rRNA amplicons of 12 taxonomically well-characterized bacterial species [21] was included every fifth lane for normalization of the fingerprint profiles using the BioNumerics software version 7.0 (Applied Maths, St-Martens Latem, Belgium). After normalization of the gels, individual bands were marked using the auto search bands option, followed by manual correction if necessary. Band intensities were calculated from the peak-area in the densitometric curves. All of the profiles were compared using the band-matching tool, and uncertain bands were included in the position tolerance settings. Bands were allocated to band-classes (Bcl) which are arbitrarily generated in a collective analysis of all 55 profiles by tracing common bands across different sample profiles. A maximum deviation of 0.5% was applied, which means that allocation of a band to a specific Bcl was only allowed if it was located at a distance less than 0.5% of the total length of the profile from the closest Bcl. Band-class designations were based on their relative position on the profile compared with the standard reference.

Taxonomic identity of Bcl was inferred by position-based comparison with a selection of previously generated clones that together represent all phylotypes identified in two cheetahs with phylogenetic clone library analysis [14]. This set of clones with known phylogenetic position was included as a taxonomic reference framework in DGGE to perform band position analysis in which a maximum deviation of 0.5% was allowed. Bands from potentially discriminating Bcl that could not be identified in this way were excised from the DGGE gel, eluted into 40μl 1x TE buffer and heated for 10 min at 65°C. Subsequently, the DNA solutions were reamplified using the same V3-16S rRNA primers as those of the community PCR. Purity of excised gel fragments was checked by comigration of bands on DGGE gels with a narrower gradient and this procedure was repeated 2–3 times until a single band was obtained. Pure bands were analyzed on a new 35–70% DGGE gel adjacent to the original faecal sample to confirm their position in the Bcl. Excised bands were sequenced using an ABI PRISM 3130xl Genetic Analyzer (Applied Biosystems) by means of the Big Dye XTerminator v.3.1. Cycle Sequencing and Purification Kit (Applied Biosystems) according to the protocol of the supplier. Sequences were obtained using both forward (F357 without GC clamp) and reverse (R518) primers, and assembled using BioNumerics software. The web-based EzTaxon Server was used to allocate sequences to species based on 16S rRNA gene sequence similarities (http://www.ezbiocloud.net/eztaxon) [22].

### DGGE data analysis

An initial exploration of similarities between DGGE profiles was performed by hierarchical cluster analysis with the unweighted pair group method with arithmetic mean (UPGMA) and using the Pearson correlation coefficient and Dice’s coefficient. Student’s t-test and Kruskal-Wallis test were used in SPSS v.21.0 for statistical comparison (α = 0.05) of the variable range in pairwise similarities between the sample sets of PL and OV and between animals, respectively. In a statistically similar manner, also the community diversity was compared using DGGE band number (species richness, S), Shannon Diversity Index (H’) and evenness (E = H’/H’_max_, where H’_max_ = lnS) as standard indices.

To assess the bacterial community stability, multiple approaches were used. First, faecal samples were binned into 15 increasing time intervals (i.e. 2 months, 4 months, 6 months, … up to 30 months between sample collection) after which the average Dice similarity coefficient across all possible sample pairs was determined in each bin. The temporal stability of the predominant faecal microbiota was then monitored by calculating the time in months between the sample dates as well as the fraction of shared Bcl between them as measured by the Dice coefficient. Next, the species richness S was plotted against time. In another approach, the evolution of the DGGE profile similarities between consecutive time points, *i*.*e*. the rate of change (Δt), was determined for each animal with moving window analysis, which was previously demonstrated to be a valuable tool for monitoring microbial community dynamics [23]. For this purpose, a matrix of similarities between the densitometric curves extracted from all DGGE patterns per animal was calculated based on the Pearson correlation coefficient. Based on the equation %change = 100-%similarity, similarity values were converted to %change values which were then plotted in a time frame of consecutive sampling points for moving window analysis. The rate of change value (Δt) was calculated as the average of the respective moving window curve data points and indicates the relative stability of the predominant microbiota within a specific animal over the study period. The higher the changes between the DGGE profiles of two consecutive sampling points, the higher the corresponding moving window curve data point will hence the higher the Δt values.

Potentially discriminating band-classes were identified by linear discriminant analysis on the total DGGE dataset with BioNumerics. In addition, quantitative information derived from relative band intensities was exported as a data matrix and exported to SPSS v21.0 for statistical analysis. Non-parametric Mann-Whitney U tests (α = 0.05) were performed to substantiate the potential discriminating band-classes in DGGE fingerprint profiles of cheetahs grouped per zoo sample set or per animal. *P*-values were corrected for multiple-testing error by using the Benjamini-Hochberg method [24].

### Real-time PCR

The choice of the four 16S rRNA gene-targeted group-specific real-time PCR assays (S1 Table) was based on the high abundance of *Clostridium* clusters I, XI and XIVa, and near absence of *Bifidobacteriaceae* previously observed in our clone library analysis of two cheetah samples [14]. In addition, also the Firmicutes to Bacteroidetes ratio was determined. Real-time PCR amplification and detection were performed in a Lightcycler480 Real-Time PCR System Instrument II (Roche). Each reaction mixture (20 μl) was composed of 10 μl 2x SensiMix SYBR No-ROX kit (Bioline), 0.3 μl of each specific primer (10 mM), 6.4 μl milliQ water and 3 μl of stock or 10x diluted template DNA. All reactions were run in triplicate and the fluorescent product was detected in the last step of each cycle. Following amplification, melting temperature analysis of PCR products was performed to determine the specificity of the PCR. The melting curves were obtained by slow heating at 0.2°C/s increments from 65°C to 97°C, with continuous fluorescence collection. The relative amount of Firmicutes and Bacteroidetes 16S rRNA in each sample was normalized to the total amount of faecal bacteria by the comparative C_T_ method [25].

For quantification of *Clostridium* clusters I, XI, XIVa and bifidobacteria, fluorescent signals were plotted against group-specific external standard curves derived from 10-fold serial dilutions of taxonomic reference cultures with known concentrations (S1 Table). All obtained standard curves met the required standards of efficiency (R^2^>0.99, 90%>E>115%) [26]. Results were transformed to log10 CFU/g wet weight faeces values.

### Real-time PCR data analysis

Data obtained with real-time PCR assays were visualized in boxplots. Student’s t-test and Kruskal-Wallis test (α = 0.05) were applied to compare the Firmicutes/Bacteroidetes ratio and mean concentrations of *Clostridium* cluster I, XI, XIVa and *Bifidobacterium* spp. between zoo sample sets and animals respectively. Following correlation analysis (α = 0.05) in SPSS, scatter plots were generated to mirror the association between target bacterial groups. Moving-window analysis was also carried out on absolute quantities (CFU/g) of *Clostridium* clusters.

## Results

### Variation and diversity of predominant bacteria from cheetah faecal samples

**Fig 1:**
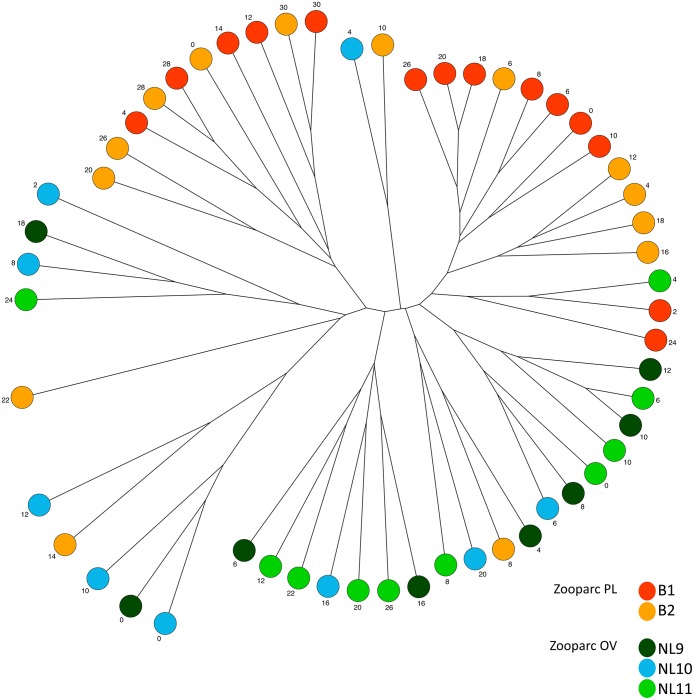
Unrooted UPGMA dendrogram based on the Dice distances matrix depicting the similarities between DGGE profiles of faecal samples collected from 5 different captive cheetahs. Samples are represented by circles, coloured by animal and labelled with number of months passed since the start of sampling.

The 55 faecal samples collected bi-monthly from 5 cheetahs over a 3-year period were initially compared by hierarchical UPGMA clustering of DGGE profiles using the Pearson and Dice coefficient. Both types of analysis did not reveal a host-specific clustering. However, samples tended to cluster together per zoo when only the presence/absence of bands in the DGGE profiles were taken into account (Fig 1). Calculation of the mean %pairwise similarity from 378 pairwise combinations for PL and 351 for OV derived from Dice/UPGMA clustering revealed that DGGE profiles of OV samples were overall significantly (two-tailed Student’s t-test, *P*<0.05) less similar (62.90% ± 0.55) and thus more variable in composition compared to the samples from PL (68.36% ± 0.47). This is also depicted by the overall longer branch distances between samples of OV in the dendrogram (Fig 1). Similar differences were also found when comparing the mean %pairwise similarity values per animal which ranged from 59.95% similarity between profiles of NL10 to 71.55% similarity between profiles of B1. Comparison of the mean %pairwise similarity values between the 5 animals revealed a significant difference (Kruskal-Wallis Test, *P*<0.05) between B2 (65.88% ± 1.08) and NL10 (59.95% ± 1.49), and between B1 (71.55% ± 1.18) and all the other cheetahs (B2 [65.88% ± 1.08], NL9 [64.08% ± 1.97], NL10 [59.95% ± 1.49] and NL11 [65.35% ± 1.48]).

As a first assessment of the population diversity revealed by DGGE, richness (S), evenness (E) and the Shannon Diversity Index (H’) were calculated for each sample. S values, expressed as the number of bands, ranged from 9 to 26 with an average of 15 bands per profile. Comparison of diversity indices identified significant differences in mean S and H’ (two-tailed Student’s t-test, *P*<0.05) between samples from PL (S_PL_ = 14 ± 1; H’_PL_ = 1.01 ± 0.02) and from OV (S_OV_ = 16 ± 1; H’_OV_ = 1.07 ± 0.03). Among the 5 animals, no significant differences were found in mean richness (S_B1_ = 14, S_B2_ = 14, S_NL9_ = 16, S_NL10_ = 16, S_NL11_ = 17) and mean Shannon Diversity Index values (H’_B1_ = 1.02, H’_B2_ = 1.00, H’_NL9_ = 1.06, H’_NL10_ = 1.05, H’_NL11_ = 1.11) (Kruskal-Wallis test, *P*_S_ = 0.191 and *P*_H’_ = 0.237).

DGGE bands across all profiles were assigned to a total of 45 different Bcl of which 31 could be taxonomically assigned using combined DGGE band position and clone-library analysis. S2 Table summarizes the distribution and taxonomic assignment of the Bcl and their relative abundances. Overall, 27 Bcl were common to all cheetahs, 8 were unique for samples from OV whereas none were unique for samples from PL. Twenty out of the 31 taxonomically identified Bcl were assigned to *Clostridium* clusters I (6 Bcl), XI (6 Bcl) and XIVa (8 Bcl), respectively. Linear discriminant analysis and statistical analysis of Bcl intensities identified 19 of the 45 Bcl to be potentially discriminating between the sample sets from PL and OV (Mann-Whitney U tests, *P*<0.05). Benjamini-Hochberg correction for multiple testing reduced this number to 9 discriminating Bcl (Table 1). Six out of these 9 Bcl were assigned to a taxonomic group by band position analysis and represented members of *Clostridium* cluster XI (Bcl 78.65 and 80.93), *Clostridium* cluster I (Bcl 77.79), the *Lactobacillaceae/Enterococcaceae* group (Bcl 38.50), *Streptococcaceae* (Bcl 32.59) and *Erysipelotrichaceae* (Bcl 39.81). Sequencing of bands corresponding to the three unidentified Bcl 28.14, 72.37 and 81.76 revealed 16S rRNA gene sequence similarity with *Eubacteriaceae*, *Peptostreptococcaceae* and *Clostridiaceae*, respectively. The strongest discriminators between PL and OV (*P*<0.01) were Bcl 77.79, Bcl 39.81, Bcl 28.14 and Bcl 81.76 (all more prominent in OV) and Bcl 38.50 (more prominent in PL). Comparison of Bcl between animals showed no significant differences after Benjamini-Hochberg correction for multiple testing (Kruskal-Wallis Test, *P*>0.05) and revealed no discriminating Bcl between animals.

**Table 1:** Distribution of discriminating band-classes between sample sets of zoos PL and OV.

		ZOOPARC PL		ZOOPARC OV			
Taxonomic assignment at family or *Clostridium* cluster level^**a**^	Bcl	Frequency (%; n = 28)	median band intensity (min-max)	Frequency (%; n = 27)	median band intensity (min-max)	*P*-adjusted^b^	Closest type strain^a^
*Clostridium* cluster I	77.79	10.7	0 (0–35.1)	51.9	31 (0–127)	<0.01	*Eubacterium multiforme* JCM 6484^T^; *Clostridium sardiniense* DSM 2632^T^
	81.76	10.7	0 (0–176.3)	59.3	181 (0–222.1)	<0.01	*Clostridium fallax ATCC 19400* ^*T*^
*Clostridium cluster* XI	28.14	14.3	0 (0–18.90)	55.6	11 (0–78.9)	<0.01	*Eubacterium tenue* DSM 6191^T^
	78.65	21.4	24.5 (0–86.7)	51.9	0 (0–56.8)	0.045	*Peptostreptococcus stomatis* W2278^T^
	80.93	82.1	142.5 (0–226.9)	85.2	214 (0–240)	0.039	*Clostridium hiranonis* TO-931^T^
*Lactobacillaceae Enterococcaceae*	38.50	78.6	65.1 (0–220)	22.2	0 (0–191.5)	<0.01	*Enterococcus cecorum* ATCC 43198^T^; *Lactobacillus sakei* DSM 20017^T^
*Streptococcaceae*	32.59	50	8 (0–89)	11.1	0 (0–127.9)	0.026	*Lactococcus piscium* CCUG 32732^T^
*Erysipelotrichaceae*	39.81	28.6	0 (0–137.6)	96.3	44 (0–160)	<0.01	*Turicibacter sanguinis* DSM 14220^T^
*Peptostreptococcaceae*	72.37	0	0 (0–0)	29.6	0 (0–89.6)	0.015	*Peptostreptococcus stomatis W2278* ^*T*^

^a^Taxonomic assignment based on a clone library analysis from faecal samples of captive cheetahs or sequencing of excised bands for Bcl 28.14, 72.37 and 81.76

^b^ *P*-values based on non-parametric Mann-Whitney U tests (α = 0.05)

### Temporal stability of the predominant faecal microbiota of five cheetahs assessed by DGGE

**Fig 2:**
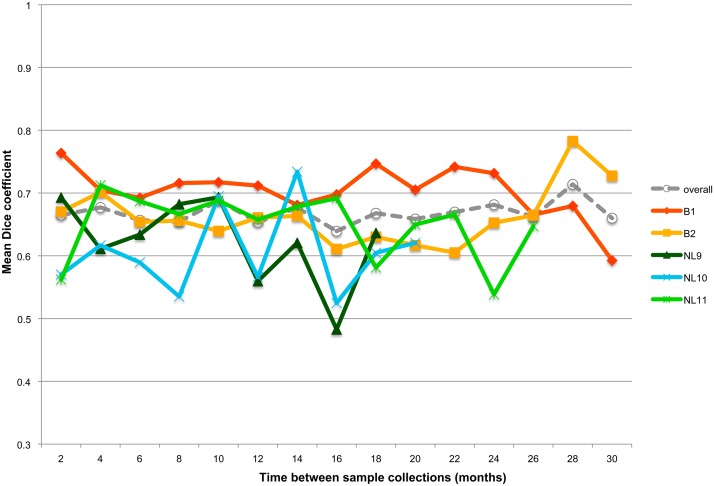
The mean Dice coefficients between samples binned into sampling intervals and depicted per cheetah.

Monitoring of shared Bcl across 15 increasing time intervals disclosed that the microbiota composition within each cheetah was relatively stable (averages between 60 and 71%), with 65.34 ± 2,06% of the same Bcl retrieved after 1 year (Fig 2). The number of Bcl shared between shorter sampling intervals was not significantly different compared to long intervals (Kruskal-Wallis test, *P* = 0.974), which indicates the persistence of a core set of species over a prolonged period of time. To define the stability of a given Bcl in function of its relative abundance, the mean sample prevalence of Bcl shared by all animals (45.45 ± 4.08% of all samples) was compared to the prevalence of non-shared Bcl (14.24 ± 2.70% of all samples). This analysis revealed a significant difference (Mannheim-Whitney U test, *P*<0.01) between both, suggesting that the more stable members of the microbiota are also the most frequently occurring ones. The mean variation in number of bands (mean S_var_), as a proxy for variation in richness, tended to be higher for animals from OV (mean S_varNL9_ = 5, mean S_varNL10_ = 6, S_varNL11_ = 5) than for animals from PL (mean S_varB1_ = 3, mean S_varB2_ = 3). The greatest S range over time was observed for NL10, which was mainly due to a single high increase in number of bands two months after the first sampling.

**Fig 3:**
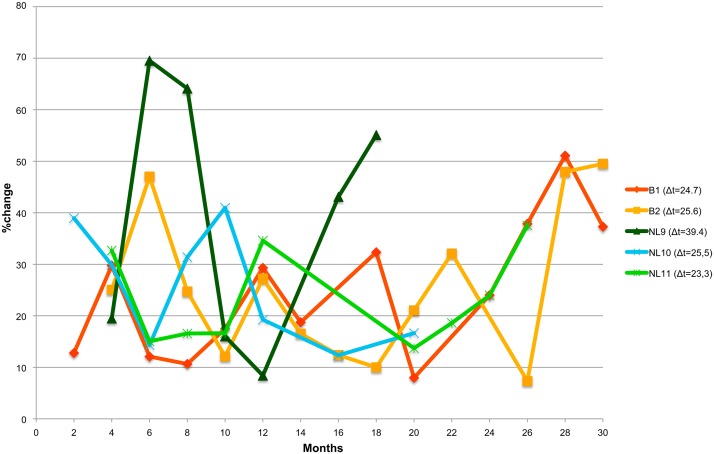
Moving-window analysis and rate of change (Δt) value of consecutive DGGE profiles per animal. Variability between month x and month x-2 was calculated from a matrix of similarities for DGGE patterns, based on the Pearson correlation coefficient.

In addition to the presence or absence of bands, their relative abundance was also considered by calculating the rate of change values (Δt) between profiles for each animal. Moving window correlation analysis for DGGE fingerprint profiles showed the highest rate of change values for NL9 (Δt = 39.4%) (Fig 3) which could be mainly attributed to an increase in %change between consecutive samples taken at the 4^th^ and 6^th^ month of sampling and the 12^th^ and 16^th^ month of sampling. Such fluctuations were not observed for the other cheetahs over the 30 months period as evidenced by lower Δt ranges (23.3–25.6%change) between consecutive sampling points.

### Real-time PCR quantification and temporal stability of *Clostridium* clusters I, XI and XIVa, bifidobacteria and F/B

**Fig 4:**
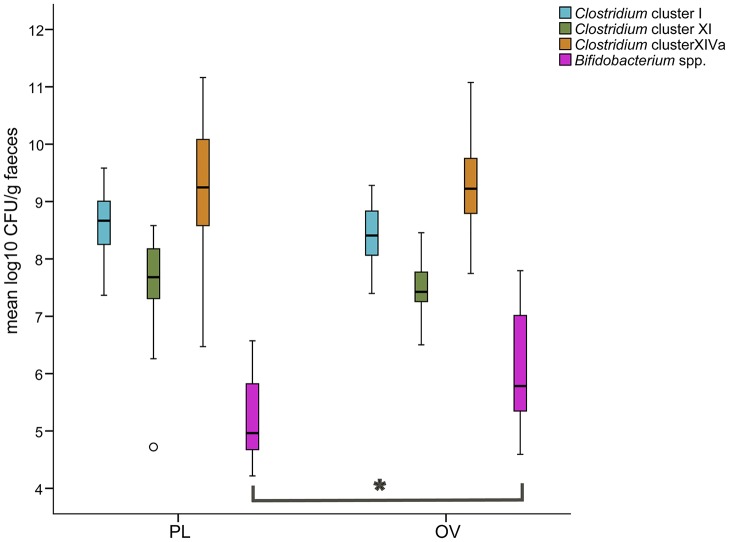
Concentrations of *Clostridium* cluster I, XI, XIVa and *Bifidobacterium* spp. in faecal samples of 2 cheetahs from Zoo Parc Planckendael (PL) and 3 cheetahs from Zoo Parc Overloon (OV), assessed by real-time PCR. Boxplots show median, interquartile range, sample minimum and maximum. The open circle indicates an outlier value for animal B2. *P<0.05, two-tailed Student’s t-test.

Members of *Clostridium* clusters XI (mean = 7.50 ± 0.09 log10 CFU/g) and XIVa (mean = 9.19 ± 0.13 log10 CFU/g) were detected in quantifiable concentrations in all 55 samples, whereas members of *Clostridium* cluster I (mean = 8.51 ± 0.07 log10 CFU/g) could not be detected in two samples of NL10 and in one sample of NL11, B2 and NL9. In agreement with these results, the DGGE profiles of these samples did not contain Bcl that were assigned to *Clostridium* cluster I. Bifidobacteria were only detected in 23 samples (42% of total), with a mean concentration of 5.59 ± 0.19 log10 CFU/g. Boxplot analysis revealed a significantly higher concentration of *Bifidobacterium* spp. in samples from OV (6.02 ± 0.03 log10 CFU/g) compared to samples from PL (5.25 ± 0.18 log10 CFU/g) (two-tailed Student’s t-test, *P*<0.05) (Fig 4). Between animals, however, quantitative ranges of the specific bacterial groups were not significantly different (Kruskal-Wallis Test, *P*>0.05).

**Fig 5:**
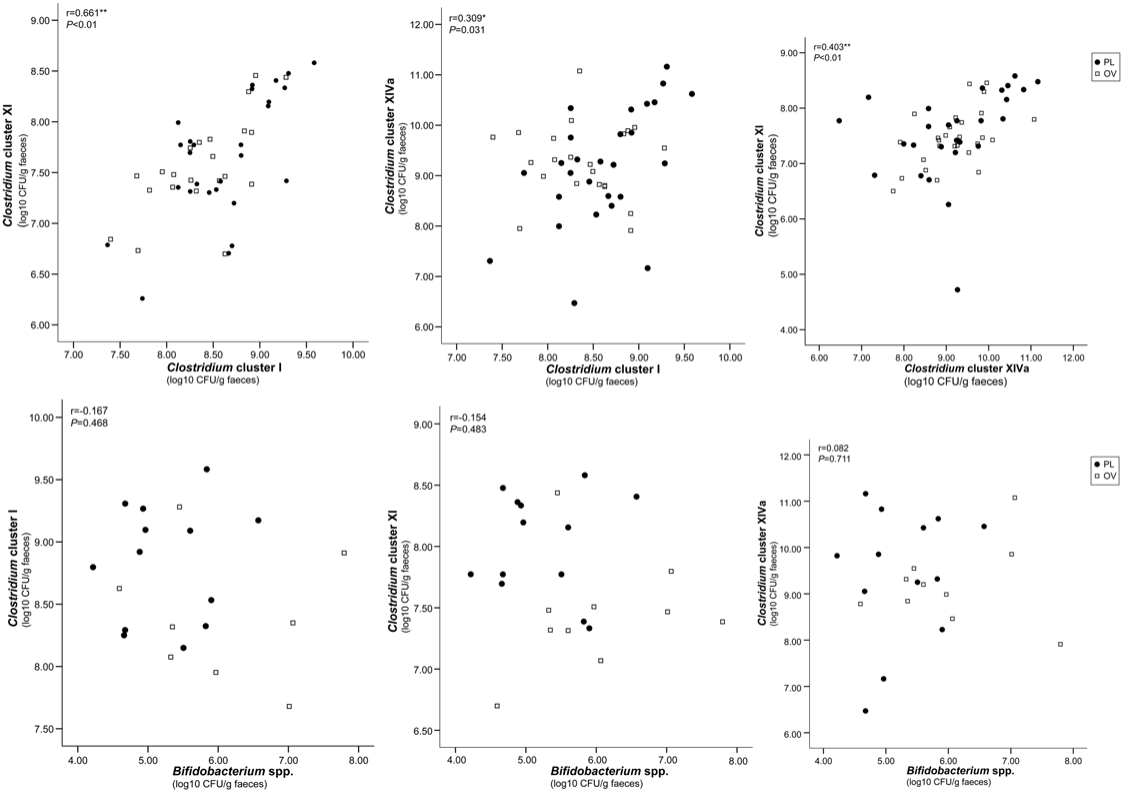
Scatter plots displaying the correlation between log10 concentrations of bacterial groups quantified with real-time PCR in captive cheetah’s faecal samples. r = Pearson correlation coefficient, *correlation significant at the 0.05 level, **correlation significant at the 0.01 level.

Correlation analysis, expressed as scatter plots, indicated a positive correlation between the relative concentrations of *Clostridium* clusters I, XI and XIVa (*P*<0.05; *P*<0.01) (Fig 5). No significant correlation was found between the relative concentrations of *Bifidobacterium* spp. and *Clostridium* clusters I (*P* = 0.468), XI (*P* = 0.483), and XIVa (*P* = 0.711), respectively.

**Fig 6:**
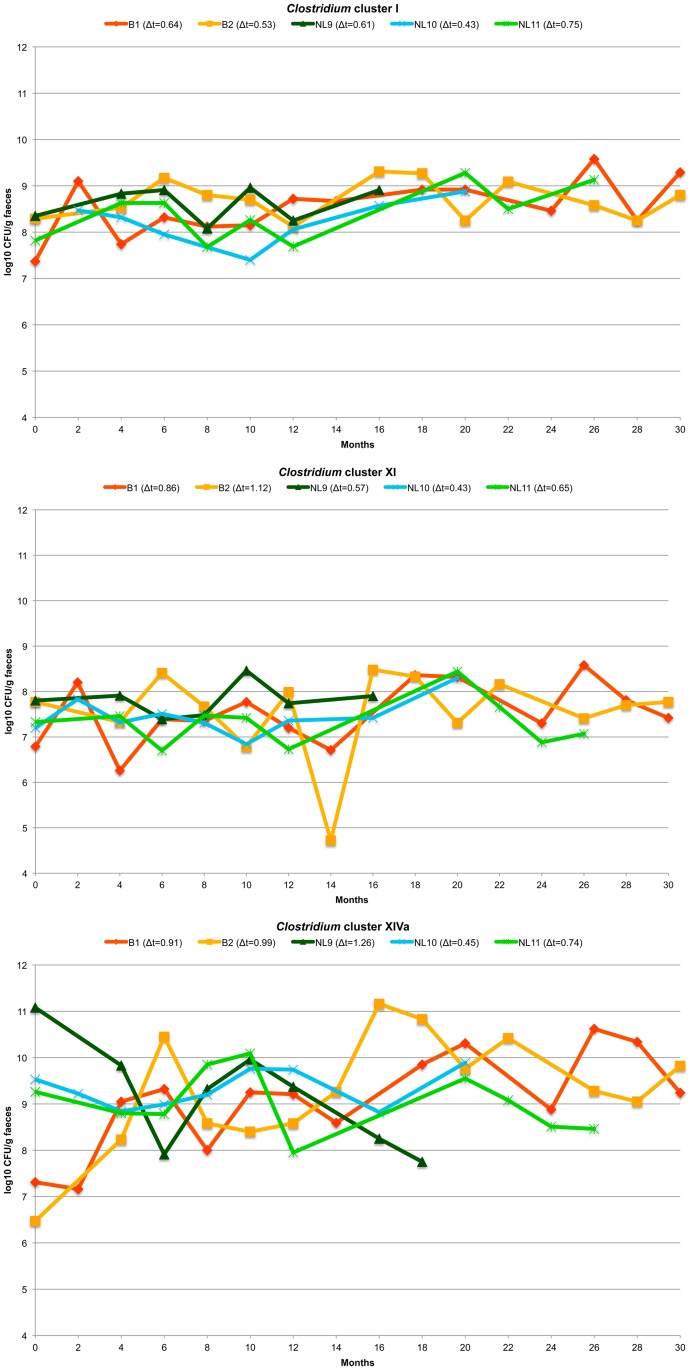
Temporal variation of *Clostridium* clusters I, XI and XIVa in faecal samples of 5 captive cheetahs, assessed by real-time PCR. Rate of change (Δt) value of consecutive DGGE profiles calculated per animal.

The temporal variation of these concentrations for each animal is shown in Fig 6 and was not significantly different between animals (Kruskal-Wallis Test, *P*>0.05). On average, overall concentrations of *Clostridium* cluster I, XI and XIVa varied over a 30 month time period with only 0.59, 0.72, and 0.87 log10 CFU/g, respectively. During the disease period of animal NL9, an increase (+0.9 log10 CFU/g) in members of *Clostridium* cluster I and a decrease (-1.9 log10 CFU/g) in *Clostridium* cluster XIVa members were observed. For the ratios of Firmicutes to Bacteroidetes, no significant differences were found between sample sets from PL and OV or between animals. However, for animal NL10 Firmicutes to Bacteroidetes ratios displayed a relatively broader range, from 0.5/0.003 to 0.8/2.2e-09.

## Discussion

The few investigations performed so far on the diversity of the cheetah gut microbiota were limited in scope as they were based on single time point samples from a total of four captive cheetahs using different taxonomic methods [1,14]. Although these studies were instrumental in providing a first cross-sectional insight in the gut microbiota composition of cheetahs under human care, it remained unclear to what extent this intestinal ecosystem exhibits temporal stability over a prolonged time period. In the present study, we monitored the predominant microbiota compositions and the quantitative abundance of selected microbiota members in five cheetahs housed in two geographically separated zoos during a bimonthly sampling campaign over a period of three years.

Our combined approach of DGGE and real-time PCR revealed that the five animals under study exhibit an overall stable faecal bacterial composition, and the inclusion of additional animals in follow-up investigations will undoubtedly help to reveal if this finding is a characteristic across the entire animal species. We found that 67% of the detected Bcl in DGGE profiles was retained over a period of 30 months, with the majority of the recurring Bcl corresponding to bacterial core phylogroups. Also at the phylum level, minor fluctuations (±2.1%) were observed in the Firmicutes/Bacteroidetes ratio. The average life span of cheetahs is 10 to 12 years, though cheetahs under human care can live as long as 20 years [27]. When taking into account the age of each animal, this means that the observed temporal stability in the study’s time frame covered four cheetahs during adulthood and one cheetah (NL9) during early aging. Our insights for cheetahs, as well as data from long-term monitoring studies of lab mice and wild chimpanzees [28,29], seem to indicate that gut microbiota stability is a general feature in mammals during adulthood. After a highly unstable early stage of microbial colonization in the mammalian GI tract during the neonatal period, complex interactions between host and resident bacteria lead towards a stable adult-like microbiota. These changes are shared among different mammalian species but differ in timing and some species-specific developmental events [30,31]. Decreased stability and shifts in diversity have been observed with advancing age in humans [32–34], and similar microbial changes were also observed in dogs [35,36]. The fact that long-term stability of gut bacteria communities is maintained during adulthood supports the concept of an ecosystem with a core community that operates in a state of homeostasis, and to a certain degree exhibits resilience to perturbations [37].

With a presence of ≥80% in the samples collected per animal over a time span of 30 months, *Clostridium* clusters XI and XIVa appear to dominate the phylogenetic core of the faecal microbiota in captive cheetahs. Together with *Clostridium* cluster I, represented in ≥50% of the samples per animal, these groups are considered relatively stable microbiota members, as evidenced by the limited fluctuations revealed by the respective qPCR assays. Monitoring studies (2–6 months) in other carnivores such as captive and wild grizzly bears also indicated that in the majority of samples the relative rank of *Clostridium* clusters I, XI and XIV determined by specific qPCR assays remained constant [11,38]. Furthermore, bacterial numbers reported for the aforementioned clusters in these studies were in the same order of magnitude (7–8 log10 CFU/g) as those obtained for captive cheetahs in the present study. For the older cheetah NL9, *Clostridium* cluster XIVa numbers were more variable, which might not only be linked to the disease period but also to an overall higher variability of this cluster upon aging, as has also been shown in humans [32].

This study confirms that in carnivores a positive correlation exists between abundances of *Clostridium* cluster I and XI [14,39]. *Clostridium* cluster I includes proteolytic and fibrolytic species, but also harbours toxinogenic members such as *C*. *perfringens* [40]. The latter species was detected in almost all faecal samples of the five cheetahs included in this study, which corroborates earlier observations suggesting that it is a common inhabitant of the cheetah’s intestinal tract [14]. *C*. *perfringens* has also been detected regularly in faeces of other carnivores [11,41–44], including domestic cats [45] and wild exotic felids [41]. Animals included in these studies were all considered healthy. In contrast, a number of case reports have described neurotoxicity, hemorrhagic enterocolitis and death in captive tigers, lions and cheetahs due to *C*. *perfringens* infections and toxins [46–48]. In non-infected animals, *C*. *perfringens* should probably be considered as a common commensal that is positively correlated to the high protein content of the carnivore’s diet [11,49]. However, further studies are needed to clarify the role of different genotypes of *C*. *perfringens* as causal agents of enterocolitis and diarrhea in carnivore species [41].

*Lactobacillaceae* are also considered to be part of the core gut microbiome of carnivores [50]. This was confirmed in the current study by their presence in ≥50% of the samples of all five cheetahs throughout the 30 months sampling period, suggesting *Lactobacillaceae* are stable members of the cheetah’s GI core microbiota. In contrast, representatives of *Carnobacteriaceae*, *Erysipelotrichaceae* and *Enterobacteriaceae* comprised most of the transient members of the cheetah’s gut microbiota. Members of the latter family have been shown to be transiently excreted in faeces of domestic cats [51]. *Bifidobacterium* spp. are considered common members of the gut microbiota in domestic cats [52], but in this study they were detected in less than half of the samples suggesting that bifidobacteria take no part in the core composition of the intestinal ecosystem in captive cheetahs.

A valid interpretation of temporal stability data benefits from additional cross-sectional analyses that may potentially link specific microbiota variations to zoo environment or animal characteristics. Pooled data analysis of overall species richness, the number of unique phylotypes derived from DGGE Bcl analysis (S2 Table) and median Bcl intensities (Table 1) indicated no significant differences at animal level, but indicated a number of differences between PL and OV sample sets that were mostly the result of variation in proportional composition at the phylotype level. At phylum level, however, the mean ratios of Firmicutes to Bacteroidetes were comparable for both sample sets throughout the study. One of the strongest discriminators that separated the two sample sets was the significantly higher prevalence of *Erysipelotrichaceae* in OV samples. In a previous study in mice, increased levels of *Erysipelotrichaceae* were linked to an increased dietary fat content [53]. Possibly, a higher dietary fat intake resulting from the more regular intake of whole prey and prey organs [54,55] by the cheetahs in OV may explain the association with higher *Erysipelotrichaceae* numbers.

No significant differences were observed upon comparison of the five cheetah’s individual microbial profiles. Likely, the minor variations observed in the predominant microbiota composition of each cheetah between consecutive sampling points reflect changes imposed by technical sensitivity and specificity and an unknown combination of intrinsic factors such as genetics, life history, diet, human and other animal exposures, and health status [56–58]. Of these, disease manifestation and subsequent therapy may be amongst the most powerful disruptors of microbiota stability as exemplified by animal NL9 that suffered from intermediate episodes of vomiting and diarrhea during the monitoring period. This cheetah was treated with Zitac vet tablets, containing the gastric acid production inhibitor cimetidine and the amoxicillin-clavulanate antibiotic Clavubactin. During that disease period, an increase in members of *Clostridium* cluster I and a decrease in *Clostridium* cluster XIVa members were observed. Upon recovery, both clusters normalized to their initial concentrations as determined before the onset of symptoms. These changes in two microbial key groups of the cheetah’s intestinal ecosystem might reflect a state of dysbiosis resulting from compromised health, but may also point to a temporary disturbance upon administration of antibiotics or a combination thereof. For instance, a significant reduction of members of *Clostridium* cluster XIVa has also been observed in the faecal microbiota of a gnotobiotic mouse model following a 7 day amoxicillin-clavulanate administration [59]. Additionally, DGGE profiles for samples from animal NL9 were characterized by a temporary decrease in species richness and change in intensity of bands, which was reflected in a higher rate of change value upon moving-window analysis. In this particular case, it appears that the relative proportions rather than the composition of the animal’s core microbiota groups were altered. It should be kept in mind, however, that the number and intensity of bands in a DGGE gel do not necessarily give an accurate picture of the microbial community due to multicopy operon heterogeneity of the 16S rRNA gene and the limited phylogenetic resolution of this technique [60–62].

Despite the limitations in detection level and taxonomic resolution, the phylogenetic information extracted from the integrated DGGE clone library approach allowed to identify a set of major bacterial lineages that may constitute the core microbiome of cheetahs under human care. Mammalian microbiota profiles tend to cluster according to the classification of their hosts into herbivores, omnivores and carnivores, which suggests that gut physiology and diet are powerful predictors of faecal microbiota composition [1,63]. However, the fact that microbiomes of conspecifics tend to resemble one another at a broader (e.g. phylum) taxonomic level does not imply that they also host similar phylotypes. This is exemplified in a recent oligotyping-based inventory of faecal bacterial communities in single samples from 68 free-ranging Namibian cheetahs [64] which revealed marked differences in some dominant phyla proportions compared to captive cheetahs. Overall, free-ranging cheetahs exhibited lower proportions of Firmicutes and higher proportions of Fusobacteria and Bacteroidetes. At the genus level, however, *Clostridium* spp. and *Blautia* spp. prevailed which further underpins their role as core microbiome members of the cheetah gut. In addition, members of the *Coriobacteriaceae* which are considered frequent residents of the feline gut [52] appeared to be enriched in free-ranging cheetahs [64], whereas this group made up only a small proportion of the clone libraries from two captive cheetahs [14]. Although these observations suggest differences in core microbial pattern between free-ranging cheetahs and captive cheetahs, microbiome studies including both zoo and wild animal populations are warranted to fully depict gut microbial diversity and stability in the strict carnivorous cheetah.

## Conclusions

The findings of this long-term monitoring study evidence that captive cheetahs host an overall stable faecal microbiota, even beyond intestinal community variations between zoo sample sets and to a lesser extent, between animals. The core of this microbiota appears to be mainly dominated by members of *Clostridium* clusters I, XI and XIVa and *Lactobacillaceae*, most of which were maintained at stable levels throughout the monitoring period. It has been previously suggested that yearly examination of a faecal sample would be sufficient to monitor changes in the intestinal microbiota composition and stability and could promote disease prevention [65]. Our finding of significant disturbances in relative proportions of key microbial groups upon disease and/or therapy in one specific cheetah seems to support this idea. For wildlife species under human care, health indices other than current faecal steroid monitoring and faecal parasite and pathogen loads may thus also prove to be useful in health surveillance.

## Supporting Information

S1 Table 16S rRNA gene-targeted group-specific primers, taxonomic reference strains and amplification programs used in real-time assays. [66–72] (PDF)

S2 Table Combined band-class and clone library analysis for 55 DGGE fingerprint profiles from faecal samples from 5 captive cheetahs. (PDF)
