# Supplementary material for: Integrated Community Profiling Indicates Long-Term Temporal Stability of the Predominant Faecal Microbiota in Captive Cheetahs
Source: PLoS One. 2015 Apr 23;10(4):e0123933. doi: 10.1371/journal.pone.0123933 (PMC4408007; doi:10.1371/journal.pone.0123933)
Supplement: S1 Table — (PDF) [file pone.0123933.s001.pdf]

Table S1. 16S rRNA gene-targeted group-specific primers, taxonomic reference strains and amplification programs used in real-time assays

Target bacterial group	Primer	Sequence (5'-3')	Taxonomic reference strains used for external standard curves	PCR program[a]	Adapted from
Clostridium cluster I	CI-F<br>CI-R	ATGCAAGTCGAGCGAKG<br>TATGCGGTATTAATCTYCCTTT	*Clostridium perfringens* LMG 11264[b]	40x (95°C-15s; 55°C-20s; 72°C-30s)	[66]
Clostridium cluster XI	CXI-F<br>CXI-R	ACGCTACTTGAGGAGGA<br>GAGCCGTAGCCTTTCACT	*Clostridium bifermentans* LMG 3029[b]	45x (95°C-20s; 58°C-30s; 72°C-45s)	[67]
Clostridium cluster XIVa	Ccocc-F<br>Ccocc-R	CGGTACCTGACTAAGAAGC<br>AGTTTYATTCTTGCGAACG	*Ruminococcus gnavus* LMG 27713[c]	40x (95°C-15s; 55°C-20s; 72°C-30s; 80°C-30s)	[66]
Bifidobacterium	g-Bifid-F<br>g-Bifid-R	CTCCTGGAAACGGGTGG<br>GGTGTTCTTCCCGATATCTACA	*Bifidobacterium adolescentis* LMG 10502[d]	40x (94°C-20s; 55°C-20s; 72°C-50s)	[68]
Firmicutes	Firm934F<br>Firm1060R	GGAGYATGTGGTTTAATTCGAAGCA<br>AGCTGACGACAACCATGCAC	n/a	45x (95°C-10s; 60°C-30s; 72°C-1)	[69–71]
Bacteroidetes	Bact934F<br>Bact1060R	GGARCATGTGGTTTAATTCGATGAT<br>AGCTGACGACAACCATGCAG	n/a		
Total bacteria	Eub338F<br>Eub518R	ACTCCTACGGGAGGCAGCAG<br>ATTACCGCGGCTGCTGG	n/a		

n/a = not applicable

[a] All amplification programs are preceeded by initial denaturation at 95°C for 10 min

[b] Grown on reinforced clostridial agar medium (CM0151, Oxoid)

[c] Grown on BBL[TM] Brain Heart Infusion medium, supplemented with 5g/L yeast extract (LP0021, Oxoid) and 5 mg/L hemin (H9039-1G, Sigma-Aldrich)

[d] Grown on modified Columbia agar [72]

n/a = not applicable
