# Supplementary material for: Integrated Community Profiling Indicates Long-Term Temporal Stability of the Predominant Faecal Microbiota in Captive Cheetahs
Source: PLoS One. 2015 Apr 23;10(4):e0123933. doi: 10.1371/journal.pone.0123933 (PMC4408007; doi:10.1371/journal.pone.0123933)
Supplement: S2 Table — (PDF) [file pone.0123933.s002.pdf]

Table S2. Combined band-class and clone library analysis for 55 DGGE fingerprint profiles from faecal samples from 5 captive cheetahs

Taxonomic assignment at family or *Clostridium* cluster level[a]	Band-class	Distribution							Closest type strain[a]
		Number of animals (n=5)	% of samples (n=55)	% of samples per animal					
				B1 (n=14)	B2 (n=14)	NL9 (n=8)	NL10 (n=9)	NL11 (n=10)	
Clostridium cluster I	64.99	*****	18,18	7	21	13	11	40	*Clostridium colicanis* DSM 2632[T]
	67.39	*****	78,18	86	79	75	44	100	*Clostridium perfringens* ATCC 13124[T]
	68.15	*****	69,09	79	79	75	33	70	*Clostridium perfringens* ATCC 13124[T]
	70.79	*****	36,36	43	57	38	11	20	*Clostridium fallax* ATCC 19400[T]
	76.09	*****	21,82	21	7	25	11	50	*Eubacterium multiforme* JCM 6484[T]
	77.79	****	30,91	0	21	63	33	60	*Eubacterium multiforme* JCM 6484[T]; *Clostridium sardiniense* DSM 2632[T]
Clostridium cluster XI	73.47	***	9,09	0	0	25	11	20	*Clostridium glycolicum* DSM 1288[T]
	74.60	*****	25,45	21	7	38	22	50	*Peptostreptococcus anaerobius* NCTC 11460[T]
	75.34	*****	23,64	21	21	25	22	30	*Clostridium glycolicum* DSM 1288[T]
	78.65	*****	41,82	71	50	13	22	30	*Peptostreptococcus stomatis* W2278[T]
	80.11	****	16,36	14	36	13	0	10	*Clostridium hiranonis* TO-931[T]
	**80.93**	*********	**83,64**	**79**	**86**	**100**	**89**	**70**	***Clostridium hiranonis* TO-931[T]**
Clostridium cluster XIVa	47.13	*****	16,36	21	7	25	11	20	*Ruminococcus torques* ATCC 27756[T]
	47.69	*****	45,45	50	21	50	44	70	*Blautia hansenii* JCM 14655[T]
	48.97	*****	47,27	36	57	50	56	40	*Blautia coccoides* JCM 1395[T]; *Blautia glucerasei* HFTH-1[T]
	50.12	*****	69,09	71	79	50	67	70	*Blautia hansenii* JCM 14655[T]; *Blautia glucerasei* HFTH-1[T]
	50.60	*****	41,82	50	36	50	67	70	*Clostridium boltae* DSM 15670[T]
	53.61	*****	56,36	71	50	50	56	50	*Blautia glucerasei* HFTH-1[T]
	**54.43**	*********	**94,55**	**93**	**100**	**100**	**89**	**90**	***Ruminococcus gnavus* ATCC 29149[T]**
	57.31	*****	54,55	57	64	75	33	40	*Coprococcus comes* ATCC 27758[T]
Lactobacillaceae	37.19	*****	29,09	7	21	50	56	30	*Lactobacillus sakei* DSM 20017[T]
	61.53	**	3,64	0	0	13	11	0	*Lactobacillus mucosae* CCUG 32732[T]
Lactobacillaceae / Enterococcaceae	38.50	*****	50,91	86	71	13	11	40	*Enterococcus cecorum* ATCC 43198[T]; *Lactobacillus sakei* DSM 20017[T]
Enterococcaceae	35.21	*****	49,09	50	21	50	89	50	*Enterococcus hirae* DSM 20160[T]
	43.67	*****	60,00	79	64	25	78	40	*Enterococcus faecalis* JCM 5803[T]
Streptococcaceae	32.59	****	30,91	64	36	0	11	20	*Lactococcus piscium* CCUG 32732[T]
Erysipelotrichaceae	39.81	*****	61,82	43	14	100	100	90	*Turicibacter sanguinis* DSM 14220[T]

Carnobacteriaceae	42.45	****	21,82	14	36	0	22	30	*Carnobacterium divergens* DSM 20623^{T}
Peptococcaceae	51.96	*	1,82	0	0	0	11	0	*Desulfonispora thiosulfatigenes* DSM 11270^{T}
Enterobacteriaceae	58.50	****	43,64	29	64	0	89	30	*Shigella flexneri* ATCC 29903^{T}
Lachnospiraceae	65.88	**	9,09	0	0	0	11	40	*Cellulosilyticum ruminicola* H1^{T}
No match	25.61	*	3,64	0	0	25	0	0	-
	27.24	***	9,09	0	0	25	22	10	
	28.14	*****	34,55	7	21	63	56	50	
	29.33	*****	36,36	36	21	38	44	50	
	30.81	****	12,73	7	0	13	22	30	
	34.77	****	16,36	7	14	38	0	30	
	41.28	*****	41,82	43	57	25	33	40	
	45.17	****	18,18	7	36	0	22	20	
	55.77	**	3,64	7	0	13	0	0	
	63.89	***	7,27	0	0	13	11	20	
	68.79	*****	10,91	7	7	13	22	10	
	72.37	***	14,55	0	0	25	11	50	
	81.76	*****	34,55	7	14	63	67	50	
	82.76	**	3,64	0	7	0	0	10	

Discriminating Bcl between zoos PL and OV are marked in grey and stable Bcl over the 3-year sampling period (>80% of the samples per animal) in bold; - = no matching clones

[a]Based on a clone library analysis from fecal samples of captive cheetahs [11]
